# Psychological reactance, misinformation, and distrust: A mixed methods analysis of COVID-19 vaccine uptake

**DOI:** 10.1017/cts.2024.15

**Published:** 2024-01-30

**Authors:** Lily Huang, Todd R. Huschka, Amelia K. Barwise, Jay-Sheree P. Allen, Wendy Wolfersteig, Kathryn Hamm, Lilliana D. Cardenas, Sean M. Phelan, Megan A. Allyse

**Affiliations:** 1 Department of Quantitative Health Sciences, Mayo Clinic, Jacksonville, FL, USA; 2 Division of Public Health, Infectious Diseases, and Occupational Medicine, Mayo Clinic, Rochester, MN, USA; 3 Robert D. and Patricia E. Kern Center for Science of Health Care Delivery, Mayo Clinic, Rochester, MN, USA; 4 Program in Biomedical Ethics Research, Mayo Clinic, Rochester, MN, USA; 5 Division of Pulmonary and Critical Care Medicine, Mayo Clinic, Rochester, MN, USA; 6 Southwest Interdisciplinary Research Center, Arizona State University, Tempe, AZ, USA; 7 Maricopa County Department of Public Health, Phoenix, AZ, USA; 8 Department of Obstetrics & Gynecology, Mayo Clinic, Rochester, MN, USA

**Keywords:** COVID-19, vaccination, psychological resistance, social determinants of health, mixed methods

## Abstract

**Background:**

Assessing perceptions of the COVID-19 vaccines is essential for understanding vaccine hesitancy and for improving uptake during public health emergencies. In the complicated landscape of COVID-19 vaccine mandates and rampant misinformation, many individuals faced challenges during vaccination decision-making. The purpose of our mixed methods study is to elucidate factors affecting vaccine decision-making and to highlight the discourse surrounding the COVID-19 vaccines in diverse and underserved communities.

**Methods:**

This mixed methods study was conducted in Arizona, Florida, Minnesota, and Wisconsin between March and November 2021, combining a cross-sectional survey (*n* = 3593) and focus groups (*n* = 47).

**Results:**

The groups least likely to report receiving a vaccination were non-Hispanic Whites, Indigenous people, males, and those with moderate socioeconomic status (SES). Those indicating high and low SES reported similar vaccination uptake. Focus group data highlighted resistance to mandates, distrust, misinformation, and concerns about the rapid development surrounding the COVID-19 vaccines. Psychological reactance theory posits that strongly persuasive messaging and social pressure can be perceived as a threat to freedom, encouraging an individual to take action to restore that freedom.

**Conclusion:**

Our findings indicate that a subsection of participants felt pressured to get the vaccine, which led to weaker intentions to vaccinate. These results suggest that vaccine rollout strategies should be reevaluated to improve and facilitate informed decision-making.

## Introduction

The novel coronavirus infection (COVID-19) was declared a pandemic by the World Health Organization in March 2020 [1,2]. As of May 2023, more than 1,125,000 people in the US have died from causes related to COVID-19 [3]. In December 2020, the Federal Drug Administration (FDA) granted an emergency use authorization for the first SARS-CoV2 vaccines, which were prioritized for healthcare personnel, long-term care facility residents, elderly individuals, and essential workers [4–7]. Three COVID-19 vaccines, including two mRNA vaccines (Pfizer-BioNTech, Moderna) and a viral vector vaccine (Janssen [Johnson & Johnson]), have become widely available since the spring of 2021. The first mRNA vaccines tested in extensive phase III trials and approved by the FDA were the Pfizer-BioNTech and Moderna vaccines. The FDA and Centers for Disease Control advised against using the Janssen COVID-19 vaccine from April 13–23, 2021, due to the possibility of developing thrombosis with thrombocytopenia syndrome (TSS). Public concerns about the safety of the COVID-19 vaccines have been sparked by the novelty of the mRNA vaccines and the TSS risk of the Janssen vaccine [8].

Many entities enacted vaccine requirements for work, school, and travel to reduce transmission and improve public safety. Amidst the mandates, vaccine and transmission safety concerns, misinformation, and politicization surrounding the COVID-19 vaccines, individuals had to decide whether to get vaccinated. One US study revealed that 22% of participants reported reluctance to receive the COVID-19 vaccine, which varied across sociodemographic traits and political affiliation, and was associated with lack of perceived threat of COVID-19 [9,10].

Studies have illustrated that the vaccine’s effectiveness against COVID-19 hospitalization during March to August 2021 was 93% for the Moderna mRNA vaccine, 88% for the Pfizer mRNA vaccine, and 83% for the Janssen vaccine [11]. Nevertheless, only 69.4% of people in the US have completed the primary series of the COVID-19 vaccines and are considered fully vaccinated as of April 5, 2023 [3].

It is important to assess perceptions of the COVID-19 vaccines to understand vaccine hesitancy and improve uptake during future infectious disease outbreaks. This mixed methods study analyzes data from a community-engaged research study developed to understand the disproportionate impact of COVID-19 on historically underserved communities. The objective of this report is to elucidate factors affecting vaccine decision-making and to highlight the discourse surrounding the COVID-19 vaccines.

## Materials and methods

The study employed mixed quantitative and qualitative methods in three phases from March to November 2021. The methodology has been described separately but is briefly stated below [12]. The study was conducted in the communities surrounding the Mayo Clinic in Arizona, Florida, and the Midwest (southern Minnesota, western Wisconsin, and northern Iowa). The qualitative phases of the study were deemed minimal risk by the Mayo Clinic Institutional Review Board (IRB 21-001802 and 21-002163). The quantitative phase was conducted by an external survey research company. The results presented here have not been previously reported.

### Survey

#### Survey instrument and sampling

The electronic survey was developed in collaboration with community members and scientific experts. The survey was distributed in English and Spanish over 8 weeks in Fall 2021 via social media links and using the contact lists of several community organizations. Participants were eligible if they were over 18 years of age and provided a primary residence zip code inside study geographic areas. A small amount of remuneration was offered to those who completed the survey.

#### Measures

Vaccine uptake intention was measured via a yes/no item, “Have YOU received at least 1 dose of a COVID-19 vaccine?.” Those responding “no” to the question were asked to complete a follow-up yes/no item: “Do you plan to receive a COVID-19 vaccine?”

Socioeconomic status was measured via an item asking respondents to describe their education level. Education is traditionally used as an indicator of SES as it is an important marker of work and economic circumstances [13]. Race, ethnicity, age, and sex assigned at birth were measured using standard survey items. Race groups with small sample sizes were combined into an “other race” group. Location was indicated by the zip code respondents entered to gain access to the survey.

#### Data analysis

Survey results were tabulated using SAS version 9.4 (SAS Institute, Inc.; Cary, NC). Completion rates varied by question. A General Linear Model (GLM) was created to compute the adjusted prevalence rates and confidence intervals of having received a dose of the vaccine and intending to receive the vaccine for each demographic group using least squared means with Tukey-Kramer adjustment for multiple comparisons. A logistic regression model was created to calculate the adjusted odds ratios and 95% Wald confidence intervals for each demographic group using White non-Hispanics, 18–29-year-olds, male at birth, highest education, and midwest location as the reference groups. Surveys with missing responses were not included in the GLM or Logistic Regression models. We examined the association between demographic characteristics and the likelihood of being vaccinated and future likelihood of vaccination while controlling for all demographic characteristics.

### Focus groups

#### Recruitment

The focus groups’ (FGs’) recruitment flyers were distributed in Spanish and English through social media and community organizations. Purposive sampling was used to recruit diverse and under-resourced populations. Recruitment for the survey and focus group sections of the study were conducted separately. Participants representing similar demographic, residential, or social communities were grouped together. Remote video conferencing sessions were conducted for up to 60 minutes. Only one FG was conducted in person due to the participants being unhoused and not having access to video technology. Focus groups included between 6 and 10 participants. A small amount of remuneration was offered to FG participants.

#### Data analysis

All FGs were digitally recorded, transcribed, and de-identified prior to analysis. FG data was organized and analyzed using the framework analytic approach by trained coders [14].

## Results

### Survey data

**Figure 1.**
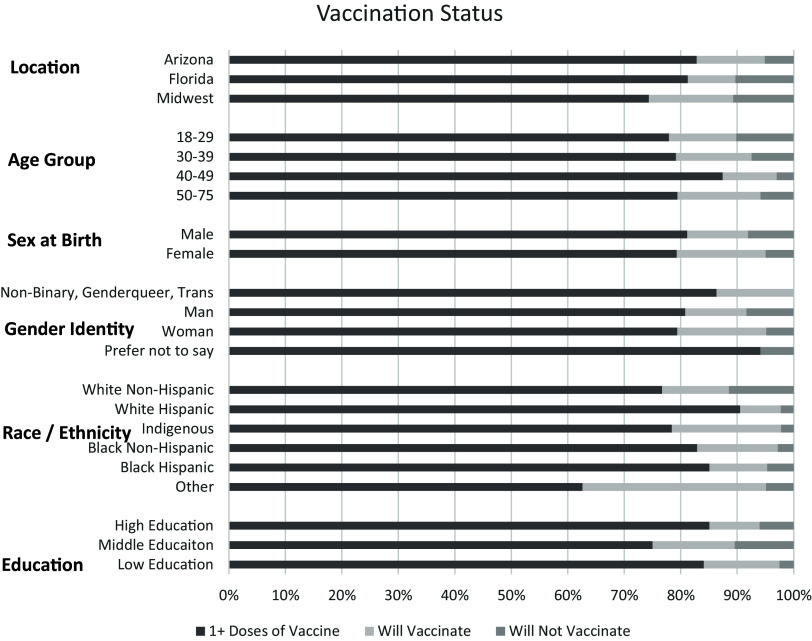
Participant demographics.

3,593 responses were included in the analyses after removal of partial completes. Participant demographics are reported in Figure 1. The association between demographics and vaccination status/vaccination intentions and shown in Tables 1 and 2.

**Table 1.** Vaccination status

		Have at least one dose of vaccine		Will not get vaccinated	
	Demographic	*p*-value	More likely	*p*-value	More likely
Location	AZ vs FL	0.73		<0.01	FL
	AZ vs Midwest	<0.01	MidWest	<0.01	Midwest
	FL vs Midwest	<0.01	Midwest	0.08	
Age Group	18–29 vs 30–39	0.89		0.09	
	18–29 vs 40–49	<0.01	40–49	<0.01	18–29
	18–29 vs 50–75	0.99		0.56	
	30–39 vs 40–-49	<0.01	40–49	0.15	
	30–39 vs 50–75	0.99		0.95	
	40–49 vs 50–75	0.38		0.99	
Race/Ethnicity	Blk Hisp vs Blk non-Hisp	0.97		0.47	
	Blk Hisp vs Indig	0.29		0.16	
	Blk Hisp vs Wht Hisp	0.28		0.95	
	Blk Hisp vs Wht non-Hisp	<0.01	Wht non-Hisp	0.09	
	Blk Hisp vs Other	<0.01	Blk Hisp	0.36	
	Blk non-Hisp vs Indig	0.68		0.97	
	Blk non-Hisp vs Wht Hisp	0.02	Wht Hisp	0.93	
	Blk non-Hisp vs Wht non-Hisp	0.04	Blk non-Hisp	<0.01	Wht non-Hisp
	Blk non-Hisp vs Other	<0.01	Blk non-Hisp	0.99	
	Indig vs Wht Hisp	<0.01	Wht Hisp	0.56	
	Indig vs Wht non-Hisp	0.98		<0.01	Wht non-Hisp
	Indig vs Other	<0.01	Indig	0.99	
	Wht Hisp vs Wht non-Hisp	<0.01	Wht Hisp	<0.01	Wht non-Hisp
	Wht Hisp vs Other	<0.01	Wht Hisp	0.82	
	Wht non-Hisp vs Other	<0.01	Wht non-Hisp	<0.01	Wht non-Hisp
M/F	Male vs Female	0.19		<0.01	Male
Education	Low vs Middle	<0.01	Low	<0.01	Middle
	Low vs High	0.94		0.95	
	Middle vs High	<0.01	High	<0.01	Middle

**Table 2.** Vaccination intentions

	Demographics	One or More Doses of Vaccine received		Plan to Vaccinate if not already Vaccinated		Will not get vaccinated	
		Yes	%	Yes	%	Yes	%
Location	Arizona	1902	82.8%	277	12.1%	117	5.1%
	Florida	347	81.3%	36	8.4%	44	10.3%
	Midwest	647	74.4%	130	14.9%	93	10.7%
Age	18-29	576	77.9%	88	11.9%	75	10.1%
	30-39	1637	79.2%	277	13.4%	154	7.4%
	40-49	612	87.4%	67	9.6%	21	3.0%
	50-75	54	79.4%	10	14.7%	4	5.9%
Sex at Birth	Female	923	79.3%	183	15.7%	58	5.0%
	Male	1964	81.2%	260	10.7%	196	8.1%
Gender Identity	Prefer not to say	16	94.1%	0	0.0%	1	5.9%
	Woman	947	79.4%	188	15.8%	58	4.9%
	Man	1852	80.8%	248	10.8%	192	8.4%
	Non-binary, genderqueer, trans	19	86.4%	3	13.6%	0	0.0%
Ethnicity / Race	Black hispanic	291	85.1%	35	10.2%	16	4.7%
	Black non-hispanic	354	82.9%	61	14.3%	12	2.8%
	Indigenous	211	78.4%	52	19.3%	6	2.2%
	White hispanic	640	90.5%	51	7.2%	16	2.3%
	White non-hispanic	1323	76.7%	204	11.8%	198	11.5%
	Other	77	62.6%	40	32.5%	6	4.9%
Education	Low education	835	84.1%	112	13.4%	21	2.5%
	Middle educaiton	1541	75.0%	224	14.5%	161	10.4%
	High education	1192	85.1%	106	8.9%	72	6.0%

White Hispanics were the most likely to be vaccinated with an adjusted prevalence of 90.7% (95%CI 86.9–94.4) and at a rate 2.95 (95%CI OR 2.23–3.91) times more likely than the reference group of White non-Hispanics. Black Hispanics and Black non-Hispanics were also statistically more likely to vaccinate while “other” were statistically less likely than all other groups with an adjusted prevalence of 55.1% (95%CI 47.3–62.9) and 0.34 (95%CI OR 0.22–0.51) times as likely as White non-Hispanics. Only the Indigenous population showed no statistical difference against White non-Hispanics. Those reporting assigned female at birth were slightly less likely than male at birth at 0.82 (95%CI OR 0.68–0.99) times as likely. Across age groups, those aged 40–49 with an adjusted prevalence of 83.5% (95%CI 80.0–86.9) were most likely and 1.88 (95%CI OR 1.39–2.53) times as likely as the reference group of 18–29. All other age groups showed no difference. Those with middle education were least likely at 71.7% (95%CI 68.3–75.1) and 0.55 (95%CI OR 0.45–0.67) times as likely as the reference group of high education. Across locations, the midwest (reference group) had the lowest vaccination prevalence at 72.7% (95%CI 68.8–77.0). Those from Arizona and Florida were 1.58 (95%CI OR 1.29–1.93) and 1.53 (95%CI 1.13–2.06) times more likely to vaccinate. Table 1 has all numerical results, including Tukey groupings for multiple comparison tests.

When asked if those that did not receive a vaccination would receive a future vaccination there were some interesting divergences from those that had been vaccinated. White non-Hispanics (reference group) were the least likely to receive a future vaccination with an adjusted prevalence of 52.9 (95%CI 44.2–61.6) while all other groups except Black Hispanics were statistically more likely to receive a future vaccination with the Indigenous population most likely at 87.4 (95%CI 73.4–100) and 7.74 (95%CI OR 3.15–18.99) times more likely. There were no statistical differences between other groups. While men were more likely than women to have been vaccinated, women were 1.58 (95%Ci OR 1.06–2.34) times more likely to report they would get vaccinated in the future. There was very little difference among age groups. Our reference group of 18–29 was now the least likely to say they would get vaccinated, but there the Tukey test for multiple comparisons did not detect a difference between any group. Those aged 30–39 did show an Odds Ratio that was significant against the reference group, which is likely due to the larger sample size between the two groups. Low Education respondents reported the highest likelihood of receiving a future vaccination with a prevalence rate of 84.9 (95%CI 74.6–95.1) and were 3.15 (95%CI OR 1.74–5.70) times more likely than High Education individuals. Those from Florida were now the least likely to future vaccinate with a prevalence of 62.2 (95%CI 50.0–74.4) and were statistically different than Arizona or the Midwest. Table 2 has all numerical results, including Tukey groupings for multiple comparison tests.

### Focus group data

A total of 47 focus groups were conducted. Focus group participants were grouped based on the attributes outlined in Table 3. In discussions, some focus group participants described hesitancy and concerns about COVID-19 vaccines, including distrust in healthcare, conflicting sources of information, and the rapid development of the vaccines.

**Table 3.** Focus group demographics

Site	Groups	Group description
Maricopa County (Phoenix area), Arizona	31†	Geography-based: NE, SE, NW, SW, Central, South Central
		Demographic-based: Latino men, Black/AA women, Parents with kids at home, Asian Americans, LGBT+, Indigenous
Duval County (Jacksonville area), Florida	7	Demographic-based: LGBT+, Black/AA women, Recently pregnant, Latina women, Cancer survivors
Olmsted County (Rochester area), Minnesota; and La Crosse County (La Crosse area), Wisconsin	9	Demographic-based: Latino men, Latina women, Young adults, Low income, Immigrant/refugee, LGBT+, Recently pregnant, Cancer survivors

† Oversampling for relative population size.

### Resistance to mandates

Many FG participants expressed that they felt they were being forced to get the vaccine due to vaccination requirements for travel, work, and school. In many parts of the country, those without proof of vaccination were restricted from accessing transportation and certain public spaces. Several people indicated that the idea of the vaccine being “mandatory” contributed to feelings that personal autonomy was being violated and deterred them from getting vaccinated.

*I am not an anti-vaxxer… My biggest fault with [COVID-19 vaccines] is the push for mandatory vaccines for travel, for kids for school, for people for jobs. In my conscience, I don't feel that is right. I feel that’s an overreach to force something like that onto people (…) I think it’s a great thing for the elderly to have the option to get the vaccine if they want it, that they can breathe a little easier. I think that’s great that it’s available, but I am fundamentally opposed to the idea of something being mandatory. For that reason, more so than any other, we would not be getting the vaccine, even if it means that we can't fly on airplanes to go visit our family.* (Arizona Moms Club Gilbert)

*I think for me the more worry is being mandatory to have to take the vaccine… I don't like to inject certain things in my body “cause I don't know what’s gonna happen to me in the next couple years.* (Florida Hispanic Women)

Others described that the stigma or “cancel culture” of not being vaccinated contributed to feelings of being forced/coerced into getting the vaccine and further deterred them from considering vaccination.

*If you have skepticism, you’re painted as a conspiracy theorist or an antivaxxer, which isn't true. Then for someone like me, I try to be very rational, know what I’m getting into, understand a full picture. That for me just turns me off even more. If you’re gonna paint me as an anti-vaxxer or conspiracy theorist, then you’ve totally lost me because I’m intelligent.* (Arizona Expectant Moms and Young Parents)

*I think… that choice [to get vaccinated] is being removed from us, and now it’s a cancel culture if you don't have the vaccine. You’re somehow an evil person, and you should just stay home, and that’s the part that I feel is not right.* (Arizona Expectant Moms and Young Parents)

### Misinformation and distrust

Some participants voiced distrust of pharmaceutical companies that developed and manufactured the COVID-19 vaccines and/or of the healthcare system more generally. Pharmaceutical companies were portrayed as lacking transparency and participating in exploitative behaviors. While some participants indicated trust in their own healthcare provider, many expressed suspicions about physicians and the healthcare system more generally.

*You had asked earlier about who we trust and about the physicians. Part of me, I feel like they — it’s a monies game with the physicians pushing these vaccines and these pharmaceutical companies working together. That’s another thought that I have when it comes to vaccines and medicine and stuff.* (Arizona South Phoenix Young Parents)

Even among individuals who were personally in favor of vaccination, many reported receiving pushback from community and family members.

*One thing that… I wasn't prepared for was my husband’s family’s distrust of the information that was coming out and distrust of the medical community… When we got vaccinated — he actually got vaccinated first — his cousins were sending messages about he shouldn't do it, and we need to be careful.* (Midwest Rochester Cancer Treatment)

Misinformation surrounding the COVID-19 vaccines included speculation that they were an active health threat, including rumors about male infertility, changes to menstrual cycles, and heart attacks.

*It’s not a natural thing for the body. It’s putting something in our bodies that can change — from what I understand, can have some real bad side effects or later on, not so much for someone older like me, but it makes me worried for my daughters or my sons as far as for reproduction, for having babies. Yeah, the miscarriages are up 400 percent and can possibly make some sterile and also that — and some of the stories that talk about how a lotta this money comes from organizations who are into population control. That makes me a little nervous.* (Arizona Seniors and Veterans)

Other participants reported hearing speculations that the vaccines contained microchip technology and unknown materials.

*Talking about it’s a microchip and they’re putting a disease in you and just all this other type of foolishness, honestly, in my opinion, which is what’s deterring other people from getting the vaccine. I think misinformation and how quickly it spreads — it seems like misinformation spreads faster than the actual facts.* (Florida Black Millennials/Black Women)

*There is something about the microchip being in the vaccine. There is rumors about COVID was made to decrease the population by try to killing all the people. There’s also about this controlling us, like those who got the vaccine can be controlled easily.* (Arizona Refugee and Advocates)

Many participants reported that the novelty of the COVID-19 vaccines and the lack of longitudinal research were a source of hesitancy that deterred some from getting the vaccine.

*I’ve definitely heard things along the lines of this vaccine has been rushed. In order to get it out quickly, they — and I don't understand the medical side of this — but I’ve just been hearing that they didn't go about creating the vaccine in the same way they have in the past and so there’s a chance that it’s — we might not be aware of some of the negative impact until after a lot of us have gotten it.* (Arizona AZCEND I-HELP)

## Discussion

The goal of this study was to examine vaccination uptake among diverse and underserved population groups and identify factors associated with decisions about getting the COVID-19 vaccine. SES was assessed primarily by education level, an established proxy metric for SES [15]. Uptake of COVID-19 vaccination was strongest among individuals of highest SES, who are more likely to understand the risk of viral transmission and infection and have accessible, high-quality healthcare [16,17]. Although some studies pose low SES as a barrier to vaccine uptake [18,19], our findings indicated comparable rates of vaccination in the highest and lowest SES groups. Individuals with lower SES may have been more likely to hold “essential worker” jobs, which were deliberately targeted for early access to vaccination, and are more likely to require vaccination as a condition of employment [20]. Many news reports also highlighted a lack of access to and uptake of vaccination in communities of color but this was not reflected in these data, which show that Hispanic and Black individuals reported higher rates of vaccination [21,22].

### Autonomy and psychological reactance

The group least likely to report vaccination uptake were non-Hispanic White males with moderate SES. It may be that this group is most susceptible to misinformation and that this contributed to an overall mistrust of either vaccines or the organizations that were perceived as “pushing” vaccines [23,24]. Psychological reactance theory posits that strongly persuasive messaging and social pressure can be perceived as a threat to freedom, encouraging an individual to take action to restore that freedom [25–28]. Vaccination mandates may have exacerbated existing hesitancies and triggered psychological reactance [24,28]. Our findings indicate that several participants felt “forced” to get the vaccine, which led to weaker intentions to vaccinate. Research also shows that White males may be more likely to display general antagonism towards authority, in particular government authority, than other groups. The significant tropes against accepting vaccination, because it was seen as being imposed on individuals in an authoritarian manner, echo the larger rhetoric of the anti-vaccination movement but also conservative messaging around freedom as an absence of obligations [29].

A study by Kriss et al. involving university students and vaccine mandates also found that uncertainty infringes upon a sense of control [30]. They found that an indirect threat to freedom created more uncertainty and thus more resistance. Their findings suggest that the uneven rollout schedule of the COVID-19 vaccine mandates may have elicited greater reactance and exacerbated unfavorable attitudes towards vaccination. Individuals in school or work institutions without a mandate during the start of the COVID-19 vaccine rollout may overestimate the restrictiveness of the potential mandate that might be put in place, causing greater reactance as they perceive a greater threat to their freedom.

By contrast, three experimental studies by Albarracin et al. found no evidence that requiring COVID-19 vaccines undermines vaccine intentions [31]. Their findings suggest that vaccine mandates strengthen vaccination intentions across groups of both high and low psychological reactance. In their study, individuals prone to high psychological reactance felt less obligated to vaccinate when required to do so, but the vaccine mandate did not decrease their motivation to vaccinate. The COVID-19 vaccines were situated in a highly politicized and uncertain landscape [32,33], which may explain reactance even amongst individuals who were originally receptive towards the vaccine in our study.

More evidence is needed to elucidate the effects of vaccine campaigns and mandates on vaccination intentions, especially in the context of political landscapes and misinformation. Our findings show that vaccine requirements may polarize some hesitant individuals away from considering vaccination altogether. Careful messaging is needed to translate the intended effect of the vaccine mandates into actual uptake [34]. Schools, businesses, and government institutions should collectively devise a vaccine rollout campaign to decrease uncertainty and encourage individuals to vaccinate. More research is needed on strategies to carefully introduce vaccine mandates to maximize uptake, including emphasis on the importance of communication, framing, and word choice [23,35].

### Distrust, misinformation, and rapid development

Among FG participants who indicated that they do not intend to get vaccinated, several cited vaccine misinformation, discussed the rapid development of the vaccines, and shared their negative perceptions surrounding healthcare systems and the government. Our findings are consistent with previous studies illustrating that these are prominent concerns affecting vaccine uptake [36–38]. Many of the participants who chose not to receive the vaccine emphasized that they were not against vaccines or “anti-vaxxers,” but were deeply uncertain due to both the novelty of the vaccine development and the misinformation surrounding the vaccines. Perceived stigma against those hesitant to vaccinate polarized these individuals further reinforcing their decisions not to get vaccinated. Conspiracy theories surrounding the vaccines created greater uncertainty, with several participants citing risk of infertility and government monitoring tactics. A randomized controlled study by Loomba et al. demonstrated that COVID-19 vaccine misinformation significantly reduced intentions to vaccinate by 6.4% in the US among those initially receptive towards the vaccines [39]. An emphasis on transparency in the development and research of vaccines, specifically mRNA vaccines, may help bolster confidence and encourage uptake.

Based on these findings, healthcare professionals and vaccine marketing campaigns might encourage vaccination via an open-ended approach, emphasizing credible sources of data and carefully explaining vaccine research to lay audiences. Open discussions may help facilitate informed decision-making and make individuals more comfortable sharing their concerns about the COVID-19 vaccines.

### Strengths and limitations

Strengths of the study include the large sample size from four states across the US. We utilized community-engaged research methods to collect data on vaccine uptake in different populations, helping communicate the lived experiences of traditionally underserved populations into the research. The use of both quantitative and qualitative methods helped contextualize our understanding of vaccine uptake among diverse groups.

Limitations include the inability to assess the response rate, sampling bias, or generalizability due to the use of social media in survey recruitment. The survey may have been less likely to be completed by people who use social media less frequently and have less experience with digital technology. Responses may have been impacted by common survey limitations, such as social-desirability bias and recall bias. People who had more favorable or unfavorable opinions and experiences might have been more inclined to take part in the study. People with limited internet access and limited English or Spanish language skills may have been less likely to respond. The qualitative FG sessions allowed for in-depth and comprehensive exploration of COVID-19 vaccine perceptions but are not generalizable.

## Conclusion

For many individuals, uncertainty surrounding the COVID-19 vaccine greatly shaped their decision about getting vaccinated. This study showed that the group least likely to report vaccination uptake were non-Hispanic White males with moderate SES, and rather than Hispanic or Black individuals. For those not getting vaccinated, this decision was influenced by vaccine misinformation, the belief that the vaccines were developed too rapidly, and negative perceptions surrounding government mandates and tactics which made them feel forced and/obligated/compelled to comply.
